# Supplementary material for: A protocol for the evaluation of a wearable device for monitoring of symptoms, and cueing for the management of drooling, in people with Parkinson’s disease
Source: PLoS One. 2023 Feb 24;18(2):e0280727. doi: 10.1371/journal.pone.0280727 (PMC9955579; doi:10.1371/journal.pone.0280727)
Supplement: S1 File — (DOCX) [file pone.0280727.s002.docx]

A Wearable Device for Cueing for the management of drooling, and monitoring of symptoms, in people with Parkinson’s

**1 Research Proposal**

The Research Proposal will be registered on the ISRCTN registry.

Saliva plays an important part in oral health maintenance, mastication, deglutition

and the start of the digestive process. It supports clear speech. Alterations to the composition and flow of saliva through hyper- or hyposecretion or anterior loss through the lips thus have potentially significant consequences (Miller et al 2019).

Sialorrhoea, also termed drooling or ptyalism, is reported as a common symptom of Parkinson’s, it is reported to be an issue in up to 70% of people with Parkinson’s (PwP), and increases in both frequency and severity as the disease progresses (Miller et al 2019). Drooling can be a major problem in PwP due to decreased automatic swallowing, particularly when people are multi-tasking and concentrating on other things such as watching television (Miller et al 2019). It is not that PwP produce more saliva than other people; however, when automatic swallows don’t occur saliva pools in the mouth leading to drooling which can be very embarrassing and restricts the social life of PwP.

Motorically sialorrhea arises from an interaction between oro-facial rigidity, lingual bradykinesia and aspects of oro-pharyngeal dysphagia. Postural, cognitive,

attentional and pharmacological factors may also contribute. Objective evaluation of sialorrhea looks at the rate and variability of flow (milliliters or milligrams per unit time; swallow intervals; consistency). Since objective measures seldom reflect patient-reported lived experience, assessment includes rating scales that capture subjective concerns.

Dysphagia in PD arises from a range of factors (Miller et al 2017). Those most pertinent to anterior drooling relate to reduced tongue motility and depressed initiation of swallow reflexes. This leads to failure to swallow saliva regularly and/or efficiently, leaving excess saliva in the mouth and the (mistaken) impression of hypersalivation.

Facial muscle rigidity can impair lip control and depress swallowing efficiency, as well as render individuals prone to anterior loss of saliva (Fereshtehnejad SM et al 2017, Kalf JG et al 2011a). Any combination of stooped body posture, flexed neck and open lips/lowered mandible may aggravate attempts to retain saliva in the mouth, hinder directing saliva posteriorly and reduce the likelihood of initiating a swallow reflex. Cognitive impairment can influence drooling likelihood and severity (Rana AQ et al 2013). The relationship may not be directly with cognitive status so much as associated changes to attention, especially during competing or dual task situations (Brodsky M et al 2012). Reynolds et al 2018 measured drooling severity and frequency at rest and during a distracting computer task in 18 people with PD reporting day-time drooling (Reynolds H et al 2018). There was no significant difference between drooling severity at rest and during distraction, but participants swallowed significantly less frequently and drooled significantly more often during the distraction task.

Current treatments decrease the production of saliva either with oral agents such as glycopyrronium, or via Botox injections into salivary glands which need to be repeated on a 3-monthly basis. These treatments are problematic, as is well known that saliva is essential for good oral health. Impaired production or loss of saliva through drooling exposes individuals to a range of negative effects including major health and psychosocial issues.

Impaired flow or consistency of saliva exposes to risks of lowered resistance to infection, depressed oral health, impaired bolus formation and transportation and implications

for digestion. Consequences include dry mouth, ulceration, tooth decay, gingivitis, candidiasis, halitosis and perioral dermatological issues (Saleh et al 2014, Barbe et al 2019, Barbe et al 2017).

As the secondary effects from drooling (eg odor, stained clothes, constant wiping) are socially undesirable in many societies, presence of sialorrhea may bring repercussions for psycho-social health of the person who drools and added burden for the carer (eg washing clothes; restricted social life) (Miller et al 2019).

NICE guidelines (NICE 2017) on the treatment for drooling problems recommended

that non-invasive treatment options, such as behavioural cueing methods, before

drug or surgical therapy is considered. Cueing has been employed to successfully

improve aspects of impaired activities in Parkinson’s, such as gait. Our research has piloted this approach using a wrist-worn device to provide haptic cues for automatic swallowing (McNaney et al 2019). In a proof of concept and feasibility pilot (McAnany et al 2019), employing a wrist-worn tactile cue to increase swallowing regularity, 22 from 28 participants found positive benefits, with significant overall group differences on visual analog scale self-rating of drooling frequency and severity pre-post the 4-week intervention. Early indications are that devices prove highly successful for many people with PD, but not everyone. Longer term follow-up has not yet been detailed. Future work is required to test out on larger populations which person, situation, cue type and frequency variables lead to more successful outcomes. Despite the limitations of our pilot study, we were able to demonstrate that the wearable haptic cueing was not only an effective treatment method, it was also a socially acceptable solution for PwP. While PD-CUE was able to discretely deliver the haptic cueing to the individual, the device itself was cumbersome and was not yet suitable for all day use, with participants wearing it for 1 hour per day only. PD-CUE was rather simplistic in design, with a single toggle switch to start and stop the haptic cueing, with no ability to modify the intervals or intensity. This required the individual to consciously, and manually, start/stop the cueing as needed and made it a less desirable everyday solution. Moreover, due to the limited functionality, we were unable to capture or understand the real-world behaviours and usage of the intervention to understand the longer-term effects.

Through this project, we will deploy the next iteration of PD-CUE, CUE BAND application (App) for use with a smartphone. The "CUE BAND" hardware is the PINE64 PineTime Smartwatch ([https://wiki.pine64.org/wiki/PineTime](https://protect-eu.mimecast.com/s/oOEUCznKEuGzOnzs4NSzA?domain=wiki.pine64.org)).  This hardware was chosen as its design is open source and it allows us to use our own firmware programming to customize the device's behaviour.  The firmware programming is a custom "fork" off the InfiniTime open source firmware ([https://github.com/JF002/InfiniTime](https://protect-eu.mimecast.com/s/0PmrCAmP2SZ5GV5C8oTS5?domain=github.com)), chosen as it provides basic watch functionality.  The primary additional features are time-based cueing schedules and device activity logs.  To support these features, wireless configuration and downloading will be implemented, and custom user interfaces added to the watch.  Additional work will be undertaken to secure the device communication, and allow the devices to be remotely updated.

The smartwatch is more discrete and comfortable to wear. It allows individuals to define a 7-day cueing schedule of when the device should start/stop cueing, as well as modify the intervals and intensity of the haptic cues. The inclusion of lifestyle monitoring sensors to CUE BAND means that we can situate and contextualise the cueing usage alongside the physical activity and sleep behaviours of PwP. We will recruit 3000 PwP to wear the device throughout our 6-week evaluation. Participants will be asked to wear the CUE BAND continuously or as much as possible during this time, giving us a greater understanding of the effectiveness and acceptability of the technology within real-world use.

CUE BAND is a small wearable device that can be worn on the wrist. Costing just £20, it has a 3-axis accelerometer (capable of a signal response up to 200Hz), Bluetooth BLE 4.0 communication, controllable vibration motor to provide haptic feedback and 70mAh battery. It also has a screen and button, as well as heart rate monitor. These hardware specifications are comparable with mainstream fitness trackers. It is the onboard software (i.e. firmware) that sets CUE BAND apart from consumer devices. Our custom firmware allows the device to be updated or reprogrammed over-the-air via a Bluetooth device such as a smartphone. This means that it is possible to change and update the functionality of any CUE BAND device remotely via the individual’s smartphone. There are no restrictions on the amount of times that the device can be updated or reprogrammed, making this an exciting device for future healthcare interventions. Currently we are exploring the application of CUE BAND to deliver haptic cueing for PwP experiencing drooling. While existing fitness trackers are able to vibrate and provide notifications, they are limited in the control over the vibration motor and those actions are typically driven by the smartphone – i.e. rely on the connection with the smartphone to instruct the fitness tracker to take action. Whereas CUE BAND has been designed as a standalone solution, meaning that once the device has been programmed with the 7-day cueing schedule, it is possible to then disconnect the smartphone (or leave it at home) and still receive the cueing prompts. All our software are Open Source technologies and available for anyone to inspect, use or repurpose. Many commercially available wearable devices are closed source or ’black-box’ technologies that obfuscate the firmware and algorithms to perform physical activity and sleep monitoring. As such, it is difficult to assess how appropriate those algorithms are for PwP and therefore how accurate the reported data are. The open source design of CUE BAND means that any publications or anonymous datasets resulting from the device can be scrutinised and validated against other open source datasets and algorithms.

We will develop a website to publish the anonymous usage data captured from CUE BAND for future research, alongside our open source software for the CUE BAND platform (wearable and mobile apps). By developing this as an open source platform we allow PwP and researchers to continue to grow and shape CUE BAND. Being open source means that anyone can use the tools we’ve created and develop new functionality or integration with the platform.

We will work with Parkinson’s UK and the Special Interest Group on Parkinson and Technology (SIGPAT), now renamed Digital Parkinson’s, to establish sustainable processes to enable researchers and PwP alike to access the device and software for personal use and future studies.

**Assessments**

Evaluation of saliva flow is challenged by a range of issues, including: difficulty obtaining objective measures in naturalistic settings: time and place variability that exists in respect of natural variation in flow rates; fluctuations in motor function experienced by PwP that can impact on swallowing and saliva control; the variety of situations for PwP concerning where they experience difficulties or not; and the subjective nature of whether an individual perceives there to be a problem present or not (Miller et al 2019). While objective measures, such as volume of saliva produced, have been used in previous clinical studies they are time consuming and costly and don’t lend themselves to research in the community. It is more appropriate to rely on rating scales.

One partially validated tool is the Sialorrhea Clinical Scale for PD (SCS-PD) (Perez-Loret et al 2007). It has seven items employing 4-point ordinal rating scales for the PwP to determine their impression of drooling over the previous week. Items cover severity and frequency when asleep and awake, impact in relation to speaking and eating, and impact on social situations. The Radboud Oral Motor Inventory for PD subscale for saliva (ROMP-S) (Kalf et al 2011b) is the only other tool currently validated for PwP. It is derived from the unvalidated Drooling Frequency and Severity Scale (DFSS-P), (Thomas-Stonell, Greenberg J et al 1988) originally drawn up for children with cerebral palsy, but employed in several other populations. It was slightly modified for ROMP-S, in particular by adding the option to score that one is troubled by (perceived) accumulation of saliva without actually drooling. The nine items, rated on 5-point ordinal scales, cover day and night-time frequency and severity, effects on speech and eating and drinking, how frequently one has to wipe away saliva, limitations on daily activity and social participation and overall impact.

Some studies have employed the drooling item in UPDRS II (Goetz et al 2008). It is a 5-point descriptive ordinal scale, ranging from 0-4; no drooling (0), excess saliva but no loss (1), night time but not awake drooling (2), awake drooling but wiping not necessary (3) and severe drooling with constant wiping/wet clothes (4). This has been criticized as a coarse scale: suggested severity levels are not spread evenly across scale-points; while it is presented as an ordinal progression, the combination of asking about three variables that may vary independently of one another (perceived amount of saliva in mouth; night-time drooling; daytime drooling) makes it difficult to classify responses accurately. The UPDRS sub-scales on facial rigidity/lip opening and motor fluctuations and their impact may provide useful supplementary diagnostic information.

**1.2 Plan of Investigation**

**1.2.1 Objectives**

1. Complete Development of CUE BAND and begin procurement.

2. Launch CUE BAND and begin user evaluation with 3000 PwP.

3. Publish the open source CUE BAND hardware and software for public use.

4. Disseminate project outcomes and publish datasets.

The project involves the deployment of CUE BAND, a novel wrist-worn technology for PwP, which has specifically been designed to provide cueing for swallowing in addition to traditional activity, sleep, and heart rate, monitoring. Within 9-months, we will complete the preparation of 3000 CUE BAND devices and publish our mobile apps (iOS & Android).

**1.2.2 Recruitment**

During the initial CUE BAND development phase of the project, we will also begin recruiting 300 PwP through the Parkinson’s UK & Digital Parkinson’s research networks and mailing lists, who meet the criteria for the cueing study.

Our inclusion criteria for the study are:

• PwP experiencing symptoms of oropharyngeal dysphagia or self-reporting as having difficulties with drooling and swallowing. See screening questions for presence of drooling problem.

• PwP that own an iOS or Android smartphone device (to support the research

engagement). Their smartphone must support Bluetooth BLE 4.0+ to communicate with the band (relatively standard in smartphones for 4-5 years).

• PwP willing and able to provide consent to participate.


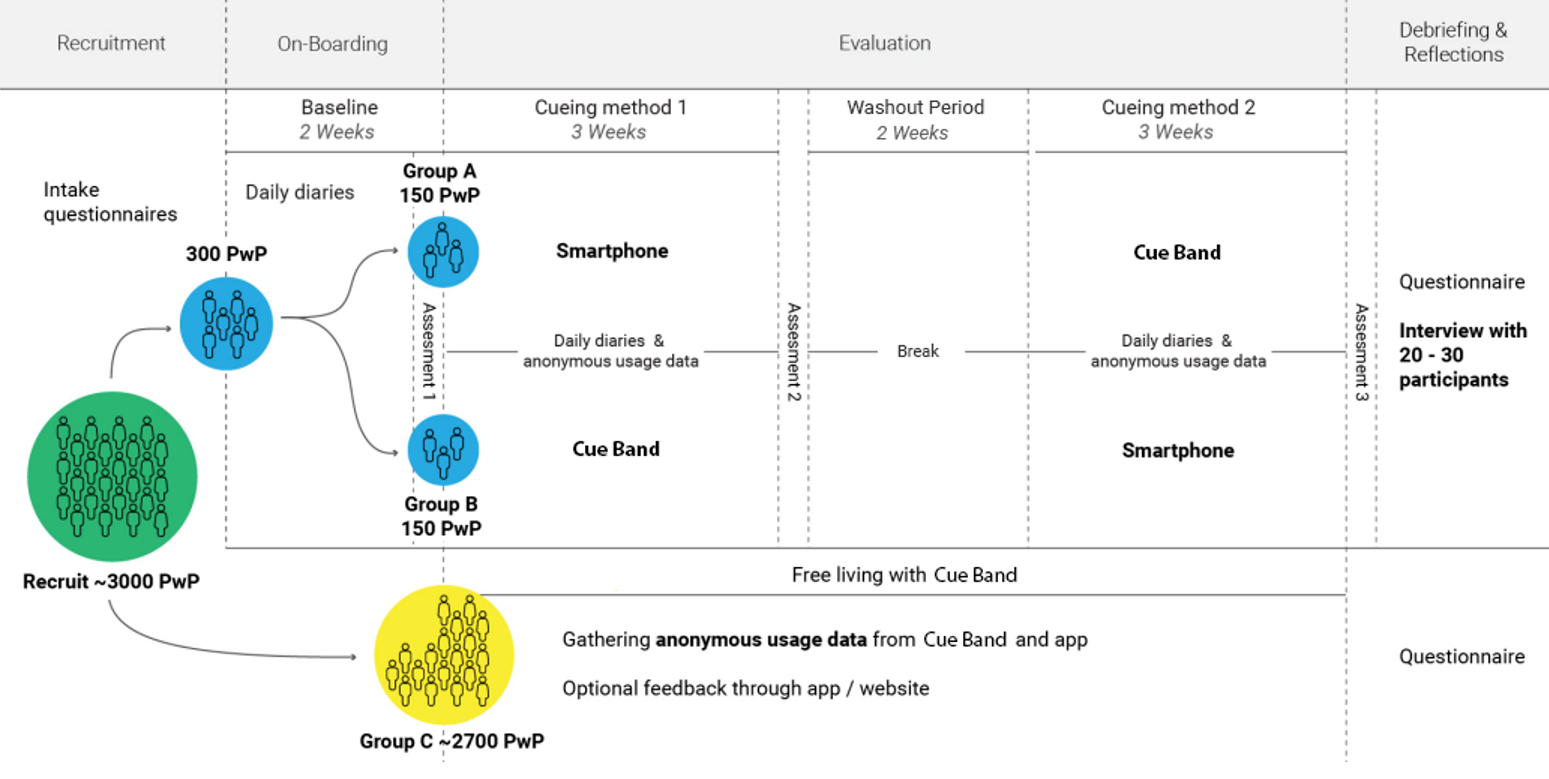


Figure 1: Overview of the cueing study recruitment and design; including

a breakdown of the study activities. NB for the cueing study we will also recruit from patients under the care of the Northumbria Healthcare NHS Foundation Trust (NHCFT) Parkinson’s Service and the North Cumbria Integrated Care Trust (NCIC) Parkinson’s Service.

**1.2.3 Study Design**

**Overall Design**

All 3,000 participants in the main study will be posted a free CUE BAND device which they can keep and continue to use beyond the study. The device will either have firmware prior to posting or the participant will be able to upload this when the device arrives. Group C, the 2700 PwP will only be providing anonymous usage data (i.e. cueing schedule, physical activity and sleep behaviours), they will also have the option to provide feedback and suggestions through the mobile app. We already have Northumbria University ethics approval for this component of the study.

**Cueing Study Design**

We will take a mixed-methods approach. Our recruitment strategy and study design are illustrated in figure 1. We will conduct a comparison study with the current state of the art assistive device – smartphone app providing notifications (audio and/or vibrations) to prompt for swallowing. Using quantitative methods, we will explore the potential effects of the cueing method (i.e. CUE BAND wearable smartwatch vs. smartphone only) on the perceived drooling severity and frequency. It is estimated there are 145,000 PwP in the UK, of whom up to 70% experience symptoms of oropharyngeal dysphagia, giving a population of 118,900. Wewill recruit 300 PwP (95%, CI=5.65%) to participate in our eight-week cueing intervention study in which we are using a within-subjects design. We will capture *formal assessments* of the individual’s symptoms before and after each intervention method. *Formal Assessments* will comprise of questions from the ROMP-Saliva (Kalf et al 2011b), the DSFS-P which looks at severity and frequency (Thomas-Stonell et al 1988), UPDRS 2.2 subset for saliva (Goetz CG et al 2008) and new non-motor symptom questionnaire (NMSQ) (Chaudhuri et al 2020), which is a modification of the first NMSQ (Chaudhuri et al 2006). The NMSQ assesses the non-motor symptoms in PD. Participants will also be asked if they have used the cueing app previously and, if so, what was their experience of it.

During this time, participants will be asked to maintain a daily diary using the tools provided through our mobile app. They will self-report on their swallowing severity, frequency and duration using visual analogue scales, placing a cross on a 100-millimetre (mm) line, (0 mm being no problem) and (100 mm being as bad as can be) (McNaney et al., 2019); as well as provide any additional comments or reflections on their experiences that day. Prior to receiving the interventions, participants will complete a two-week baseline during our on boarding phase, whereby they complete the daily diary (drooling severity chart) and self-reporting without any intervention.

For the cueing intervention component of the study, as well as recruiting participants from the 3,000, we will also recruit from Northumbria Healthcare NHS Foundation Trust (NHCFT) Parkinson’s Service, and from the North Cumbria Integrated Care Trust (NCIC) Parkinson’s Service (for which Professor Richard Walker is the current clinical lead), who meet inclusion criteria for the study, i.e. have PD, report a problem with drooling, and are cognitively able to complete the study. They will be guided to the website to register for the study, with the assistance of research staff if necessary.

For our 300 PwP involved in the cueing method intervention study (Groups A & B) each participant of Groups A & B will experience both interventions (CUE BAND wearable and smartphone only); however, the intervention order will be counter-balanced to avoid learning effects or additional bias. Intervention order will be based on a randomised allocation. They will be asked to use the intervention for a period of three weeks, while maintaining the daily diaries and self-reporting. After 3 weeks, the participants will have two weeks of no intervention. The cueing methods will be deactivated for this time period. Then the participants will receive the alternative intervention for three weeks. The Cue Band method will be disabled when users are using the Smartphone only. Following the intervention phase of our study, participants will be asked to complete a questionnaire about their experience and preferences regarding both interfaces (CUE BAND wearable and smartphone only). Participants will complete an adapted system usability scale (Bangor, Kortum & Miller, 2008). Participants will be allowed to retain the CUE BAND wearable beyond our evaluation; however, we will disable any data sharing with the research team, unless they give permission for any further time limited data to be collected. As part of the debrief phase we will assist the participants in setting up their preferred prompting method (CUE BAND wearable or smartphone only), or disabling and removing the mobile applications if they no long wish to use them.

A subgroup who express an interest, will enter a 3-week phase at the end of the interventions, when they receive neither intervention to see if there is a carry over effect. They will then have the same follow-up assessments as at the end of the intervention period prior to exiting the study.

Through qualitative methods, we hope to understand daily experiences of PwP with symptoms of drooling and their opinions on the acceptability and feasibility of personal assistive technologies to cue for swallowing. We will aim to recruit until data saturation from the intervention study (Groups A & B) to participate in a subsequent semi-structured interview (in person or via Telephone or Online audio/video call).

**1.2.4 data collection measures**

All 300 participants will be invited to share their anonymous usage data from the CUE BAND and companion mobile app. Their data comprise:

• Cueing Schedule: pre-programmed daily schedule for the cueing periods

• Cueing Usage: any additional manual activation or deactivation of the cueing beyond the pre-programmed behaviours.

• Physical Activity: time-banded levels of physical activity and movement captured from the CUE BAND accelerometer.

• Sleep behaviours: time-banded measurements of physical movements during sleeping captured from the CUE BAND accelerometer – can be mapped to sleep phases / levels.

Participants will need to have sufficient cognitive function and manual dexterity with / without assistance from carers. Participants will be asked to provide self-reported *formal assessments* of symptoms before and after receiving cueing method interventions. They will complete basic demographic information (age & sex) and PD related information (e.g. time since diagnosis). They will have assessment with the ROMP-Saliva (Kalf et al 2011), UPDRS 2.2 subset for saliva (Goetz CG et al 2008), NMSQ (Chaudhuri et al 2020) and PDQ-8 (Jenkinson et al., 2007) captured through the mobile app interface. In addition to these assessments, participants will also be asked to provide daily diary entries – Likert-scale reporting of each of the factors of interest:

• Frequency: to indicate the number of separate episodes of drooling they experienced that day.

• Duration: to indicate how long those individual episodes lasted.

• Severity: an indication of the perceived impact of the episodes.

Finally, participants will have the option to provide additional comments and reflections through text input or audio recording in the smartphone app at any time. These will be qualitatively analysed to report on the experiences of PwP using the cueing interventions. We will conduct semi-structured optional interviews on a sub-sample of participants who consent.

1.2.5 Consent procedures

For the larger study of 3,000 people will register their interest online, following which they will be sent a “patient information sheet” (PIS) via the app and will provide consent via an online form, with opportunities to reconfirm consent.

Similarly, for the cueing study participants will be able to provide consent via an online form, having received the PIS via the app. Participants will also have the option to complete face-to-face consent if they are attending a clinic appointment, consent via the app or they have the option for postal or witnessed consent. For those who opt for postal consent, a researcher will mail a copy of the consent form to the participant. A telephone or video call will be scheduled to talk the participant through the consent form and the form will be signed by the participant. Once completed, the participant will return the originally completed consent form back to be signed by the researcher. There will be a discrepancy in dates between the participant and researcher signatures.

For witnessed consent, a researcher and independent witness will be present during the signing of the consent form over a telephone/video call. The researcher will ask the participant if they agree with the points addressed on the consent form with the witness present. Following agreement from the participant that they are happy to proceed, the researcher and witness will sign and date the consent form attesting that the requirements for informed consent have been agreed by the participant.

All participants will be provided with a copy of their completed consent form, either in paper or digital format.

**Sample size calculation for PD drooling study**

Sample size calculation: Difference between the two groups in the change in ROMP saliva score from baseline to follow up is the primary outcome of interest. However, a minimal clinically important difference (MCID) for this change has not been established. In a recent study Mestre et al (2020) identified an MCID of 3 (reduction of 1 point in more than 2 items) for the ROMP-saliva score based on clinical judgement. In their interventional study the ROMP-saliva scores were; 22.7 (standard deviation (SD) 5.5) at baseline and 16.5 (SD 5.7) at follow-up in the intervention group.

Although this is a crossover trial we do not expect a substantial cross over effect, with the impact of the intervention largely stopping as soon as the intervention stops. However, we will investigate the presence of a crossover effect during the analysis. Nevertheless, we have allowed for a cross over effect by assuming a design effect of 1.4, which is likely to be at the upper end of any effect. We have also assumed a loss to follow-up of 30%. Although this may seem small, studies involving PD patients tend to have relatively low drop-out rates (Harvey et al, 2019).

Setting α = 0.05; β = 0.10 (90% power), MCID at 3 and assuming the SD for the change in score to be no greater than the SD for the baseline score (5.5), suggests a sample size of 71 per group (142 in total). Allowing for a design effect of 1.4 and loss to follow up of 30%, suggests a minimum sample size of 284.

**Data Analysis –**

We will Researchers conducting data analyses will be blinded to the group allocation. We will analyse drooling frequency and severity before and after the intervention phases. The primary outcome measure will be changes in the ROMP-Saliva. Secondary outcome measures will include changes in frequency, severity, and duration of drooling on the visual analogue scales, UPDRS 2.2 subset for saliva, NMSQ and PDQ-8.

The primary analysis will be a comparison of change from baseline of ROMP-saliva score for the two intervention groups. Data for the two groups will be compared using standard descriptive statistical (e.g mean, SD) initially. Mixed effects modelling will be used to compare outcomes with adjustment for cross over and any other relevant covariates where residual differences are not fully accounted for through randomisation.

The semi-structured interviews will be analysed using Braun and Clarks (2006) six-step approach to Thematic Analysis.

**References**

Bangor A, Kortum PT, Miller JT. An empirical evaluation of the system usability scale. Intl. Journal of Human–Computer Interaction. 2008 Jul 29;24(6):574-94.

Barbe AG, Ludwar L, Scharfenberg I, et al. Circadian rhythms and influencing factors of xerostomia among Parkinson’s disease patients. Oral Dis. 2019;25(1):282–289. doi:10.1111/odi.12942

Barbe AG, Bock N, Derman SHM, Felsch M, Timmermann L, Noack MJ. Self-assessment of oral health, dental health care and oral health-related quality of life among Parkinson’s disease patients. Gerodontology. 2017;34(1):135–143. doi:10.1111/ger.12237

Braun, V., & Clarke, V. Using thematic analysis in psychology. Qualitative research in psychology. 2006; *3*(2), 77-101.

Brodsky M, Abbott K, McNeil M, Palmer C, Grayhack J, Martin-Harris B. Effects of divided attention on swallowing in persons with idiopathic Parkinson’s disease. Dysphagia. 2012;27(3):390–400. doi:10.1007/s00455-011-9381-x

Forbes, Susanne Tluk, Vandana Dhawan, Annette Bowron, Adrian J. Williams, and Charles W. Olanow. International multicenter pilot study of the first comprehensive self completed

nonmotor symptoms questionnaire for Parkinson’s disease: The NMSQuest study. *Movement Disorders*, 21(7):916–923, 2006.

Chaudhuri KR, Schrag A, Weintraub D, Rizos A, Rodriguez-Blazquez C, Mamikonyan E, et al. The Movement Disorder Society Nonmotor Rating Scale: Initial Validation Study. Movement Disorders. 2020;35(1):116-33.

Christopher G Goetz, Barbara C Tilley, Stephanie R Shaftman, Glenn T Stebbins, Stanley Fahn, Pablo Martinez-Martin, et al. Movement Disorder Society UPDRS Revision Task Force. Movement Disorder Society sponsored revision of the Unified Parkinson’s Disease Rating Scale (MDS􀀀UPDRS): Scale presentation and clinimetric testing results. *Movement Disorders*, 23(15):2129–2170, 2008. doi:10.1002/mds.22340

Fereshtehnejad SM, Skogar O, Lokk J. Evolution of orofacial symptoms and disease progression in idiopathic PD: longitudinal data from the Jonkoping Parkinson registry. Parkinsons Dis. 2017;2017:1–8. doi:10.1155/2017/7802819

Harvey M, Weston KL, Gray WK, et al. High-intensity interval training in people with Parkinson's disease: a randomized, controlled feasibility trial. *Clin Rehabil* 2019; **33**(3): 428-38.

Jenkinson C, Fitzpatrick R, Peto V, Greenhall R, Hyman N. The PDQ-8: development and validation of a short-form Parkinson's disease questionnaire. Psychology and Health. 1997 Dec 1;12(6):805-14.

Johanna G. Kalf, George F. Borm, Bert J. de Swart, Bastiaan R. Bloem, Machiel J. Zwarts, and Marten Munneke. Reproducibility and Validity of Patient-Rated Assessment of Speech, Swallowing, and Saliva Control in Parkinson’s Disease. *Archives of Physical Medicine and Rehabilitation*, 92(7):1152–1158, 2011b.

Kalf JG, Munneke M, van Den Engel-Hoek L, et al. Pathophysiology of diurnal drooling in PD. Mov Disord. 2011a;26(9):1670–1676. doi:10.1002/mds.23720

Kallol Ray Chaudhuri, Pablo Martinez, Martin, Anthony H.V. Schapira, Fabrizio Stocchi, Kapil Sethi, Per Odin, Richard G. Brown, William Koller, Paolo Barone, Graeme MacPhee, Linda Kelly, Martin Rabey, Doug MacMahon, Sue Thomas, William Ondo, David Rye, Alison

National Institute of Clinical Excellence (NICE). Parkinson’s disease in adults: (NICE) guideline NG71. London, GB: NICE; 2017.

Nicholas Miller, Margaret Walshe, Richard Walker. Sialorrhea in Parkinson’s disease: Prevalence, impact and management strategies. Research and Reviews in Parkinsonism March 2019; (9): 17-28. DOI: 10.2147/JPRLS.S177409

Mestre TA, Freitas E, Basndwah A, et al. Glycopyrrolate Improves Disability From Sialorrhea in Parkinson's Disease: A 12-Week Controlled Trial. *Mov Disord* 2020; **35**(12): 2319-23.

Miller N. Swallowing in Parkinson’s disease: clinical issues and management. Neurodegener Dis Manag. 2017;7(3):205–217. doi:10.2217/nmt-2017-0006

Perez-Lloret S, Pirán-Arce G, Rossi M, Caivano-Nemet ML, Salsamendi P, Merello M. Validation of a new scale for the evaluation of sialorrhea in patients with PD. Mov Disord.

2007;22(1):107–111. doi:10.1002/mds.21152

Rana AQ, Khondker S, Kabir A, Owalia A, Khondker S, Emre M. Impact of cognitive dysfunction on drooling in Parkinson’s disease. Eur Neurol. 2013;70(1–2):42–45. doi:10.1159/000348571

Reynolds H, Miller N, Walker R. Drooling in Parkinson’s disease: evidence of a role for divided attention. Dysphagia. 2018;33 (6):809–817. doi:10.1007/s00455-018-9906-7

Roisin McNaney, Nick Miller, John Vines, Patrick Olivier, Karim Ladha, Daniel Jackson, and Richard Walker. The feasibility and acceptability of using a novel wrist worn cueing device to self-manage drooling problems in people with Parkinson’s disease: A pilot study. *Journal of Rehabilitation and Assistive Technologies Engineering*, 2019: 6:11/2055668319852529

Saleh J, Figueiredo MAZ, Cherubini K, Salum FG. Salivary hypofunction: an update on aetiology, diagnosis and therapeutics. Arch Oral Biol. 2015;2:242–255. doi:10.1016/j.archoralbio.2014.10.004

Thomas-Stonell N, Greenberg J. Three treatment approaches and clinical factors in the reduction of drooling. Dysphagia. 1988;3 (2):73–78.

**Model questions for case history taking for drooling in PD**

Impairment level:

Do you have trouble with loss of saliva?

Do you loose saliva during the night (have a wet pillow)?

Do you have trouble with loss of saliva when you’re speaking?

Do you have trouble with loss of saliva at mealtimes?

Do you choke on your saliva?

Do you have trouble with mucus/phlegm in your throat or do you often have to cough/clear your throat?

Activity level:

What do you do to remove saliva that is running out your mouth ; do you use a handkerchief, and if so how many a day?

Have you been restricted in your activities by the drooling/loss of saliva?

Participation level:

Have you been affected in your social contacts by the drooling?

How far is your drooling/loss of saliva a worry/annoyance to you?

**DSFS-P Severity and frequency of drooling in PD (Thomas-Stonell et al 1998)**

During the day when you’re awake do you experience drooling/loss of saliva?

-No, during the day I don’t experience drooling and I don’t have the feeling of having too much saliva/spit in my mouth.

- No, during the day I don’t experience drooling, but I do have the feeling of having too much saliva/spit in my mouth.

-Yes, Yes, during the day I have some drooling/saliva around the corners of my mouth or a bit over my chin.

Yes, I experience drooling during the day and you can see it on my clothes.

Yes, I experience drooling during the day and you can see it on books, on the floor or around other places in the house.

Frequency:

How often are you troubled by drooling during the day?

-Almost not at all, less than once a day

-Now and again: average/round about once or twice a day

-Frequently, two to five times a day

-Often: six to ten times a day

-Pretty well constantly

**Radboud Oral Motor Inventory for PD (ROMP-saliva) (Kalf et al 2011b)**

Always circle the closest answer

(for severity see DSFS-P above)

When are you especially troubled by loss of saliva?

(e.g. whilst reading; looking at TV, cycling, bending forwards)

*Do you experience loss of saliva at night (or in the day when you have a nap)?*

No, I don’t dribble/lose any saliva at night

Yes, I have a wet pillow now and again at night

Yes, I regularly have a wet pillow at night

Yes, every night my pillow and some of the other bedclothes are wet

*Does (loss of) saliva hinder you when you’re eating and drinking?*

No, (loss of) saliva doesn’t hinder me when I’m eating and drinking

Yes, (loss of) saliva hinders me somewhat when I’m eating and drinking

Yes, (loss of) saliva hinders me regularly when I’m eating and drinking

Yes, (loss of) saliva hinders me often when I’m eating and drinking

Yes, (loss of) saliva hinders me all the time when I’m eating and drinking

*Does (loss of) saliva hinder you when you’re speaking?*

No, (loss of) saliva doesn’t hinder me when I’m speaking

Yes, (loss of) saliva hinders me somewhat when I’m speaking

Yes, (loss of) saliva hinders me regularly when I’m speaking

Yes, (loss of) saliva hinders me often when I’m speaking

Yes, (loss of) saliva hinders me all the time when I’m speaking

*What do you have to do to wipe away the saliva?*

I don’t have any saliva to wipe away

I always keep a handkerchief with me to wipe it away

I need one or two handkerchiefs a day to wipe away the saliva

I need more than two handkerchiefs a day to wipe away the saliva

I have to wipe away so often that I always need tissues or kitchen paper in my hand or I have to use a towel to protect my clothes

*Does (loss of) saliva hinder/bother you in your contact with others?*

No, (loss of) saliva doesn’t hinder me in my contact with others

No, I have to pay more attention when I’m with others, but that doesn’t hinder me

Yes, I have to pay a lot of attention when I know that others can see my loss of saliva/dribbling

Yes, I try to avoid contact when I’m having trouble with (losing) saliva

Yes, I often notice that others avoid contact with me

*Does (loss of) saliva restrict you in your activities (work, hobbies)?*

No, (loss of) saliva doesn’t hinder me in my activities

No, I have to pay more attention when I’m busy/occupied, but that doesn’t hinder me

Yes, I have to pay a lot of attention when I’m busy/occupied and that costs extra effort (e.g. cleaning things up)

Yes, I am clearly limited in my activities by my loss of saliva

Yes, there are lots of activities that are important to me that unfortunately I can’t do any more

*How far does (loss of) saliva worry/annoy you?*

Not applicable, I don’t have any difficulties with that

Losing saliva worries me a bit

I find the loss of saliva fairly troubling, but there are worse things

Loss of saliva worries/ annoys me a lot because I have a lot of trouble with it

I find loss of saliva the worst thing about my Parkinson’s
