# Supplementary material for: A protocol for the evaluation of a wearable device for monitoring of symptoms, and cueing for the management of drooling, in people with Parkinson’s disease
Source: PLoS One. 2023 Feb 24;18(2):e0280727. doi: 10.1371/journal.pone.0280727 (PMC9955579; doi:10.1371/journal.pone.0280727)
Supplement: Attachment — Submitted filename: Response to reviewers.docx [file pone.0280727.s003.docx]

| Reviewer 1 comments | Response |
| --- | --- |
| The quality of the technical English is appropriate, but corrections will be needed. I suggest that the authors undertake a thorough round of copyediting. For example, see Lines 50, 59, 62, and 74. - | We’ve made edits throughout the document to address grammatical issues and syntax errors. |
| The authors claim no conflicts of interest, but a device and application are being tested here and the authors describe these using the third person possessive pronoun. It is currently free, but will it always be so? Even if it were free, are there no commercial implications of the resulting data that this trial will produce? What commercialisation options are being explored through this work by the participating universities? The authors need to be more forthcoming about these issues instead of making standard declaration by ticking a box. | We can confirm that this application is free and will remain free beyond the project. This has been a strong motivation for the technical approach and design of Cue Band and the research team are passionate about ensuring that any innovations that result from this research remain in the public domain. As such we wrote in detail in the funding application about our plans to Open Source all of the hardware and software developed during this research. This has been signed and agreed by all of the participating institutions, therefore we are confident that we will not experience any difficulties in making these technologies or datasets available for future use.  We have intentionally adopted open source licenses that would not prohibit future commercialisation of the technology as this might be necessary to support access and availability in the future. However, we firmly believe that this is a role best served by organisations like Parkinson’s UK. To that end our plans at the end of the study are to transfer ownership to Parkinson’s UK, of the smartphone apps from the in Google Android Playstore & Apple iOS AppStore.  While we’ve not written in this level of detail the protocol paper, we have certainly tried to communicate our intentions and address any potential concerns around conflicts of interest with respect to the technologies developed or datasets created.  on Page 4, in the section ‘The Cue Band Project’ we specifically state that Cueband is ‘an open source wrist-worn technology’.  We do also repeat this information with the ‘Overall Study Design’ section where we specifically list ‘Publish the open source cueband hardware and software for public use’ as one of our objectives.  Aggregated and anonymous datasets will be published and made available for future research use. Again, this was listed as one of our Objectives of the Evaluation phase – ‘disseminate the project outcomes and publish datasets’. |
| Formally, the condition is called "Parkinson's Disease" or "Parkinson Disease". It is most certainly **not** "Parkinson's". This adoption of the colloquial trivialises the condition in my view. | Changed to Parkinson’s Disease (PD) throughout the manuscript. |
| I cannot find a description of the part of the study involving 3,000 participants. | We’ve added additional text to section ‘the cue band project’ that describes the role of the remaining 2700 participants in group C of our study. |
| I am unclear whether participants will be limited to those in the UK. The website and applications are potentially accessible from around the world. This needs to be clarified. | Currently only participants in the UK will be recruited to the study.  While the website is indeed accessible globally, it is only possible for participants to join the study via our smartphone applications. We have limited their access / visibility in the corresponding app stores to only appear for individuals registered to the UK app stores.  Furthermore, our study requires participants to provide a home postal address for delivery of the Cue Band device. Again, we have limited this to only accept valid UK postal addresses. Therefore, we are confident that our participants are indeed from the UK only.  Despite our efforts to restrict the study to UK participants only, we have in fact designed and developed this technology to be made available beyond the UK. This includes specific technical efforts within the smartphone applications to ensure that internationalisation and translation is possible. We hope to begin this process next year with the support of our Portuguese collaborators and start to open up access to other global app stores and users. This desire has also shaped our approach to the production of the Cue Band device itself, which is entirely open source hardware and software – meaning that it can be manufactured by anyone, anywhere. Ultimately we hope these decisions we help to ensure that no one is excluded from accessing the technology. |
| How will you ensure that participants, especially those with dementia, are mentally competent to provide consent? | If they were able to install the mobile application and complete the consent, demographic, and postage questionnaires in order to receive the CueBand we would assume they are mentally competent to provide consent.  Is there are better suggestion for how we would handle such checks in this type of large-scale and remote participation study? |
| Justification for the sample size is missing and must be provided. | This has been added the manuscript. |
| The authors have not provided information on the exploration of safety or adverse events outcomes. This is an important omission. | As now detailed in the text, “Participants will be encouraged to report any safety issues or adverse events as soon as possible via the app or, if necessary, by phone or email. These will be followed up as soon as possible and, if there are implications for other participants, these will be shared via the app”. |
| Line 325: What do the authors mean by "crossover"? They must list the covariates that will be used in the adjustments. Alternatively, they must prespecify the definition that they will use to judge whether a covariate is to be used in the adjustment or not. | We have provided additional details within the manuscript regarding this. |
| The authors must specify a level of significance. They must report that they will present confidence intervals. | The level of significance will be set at P<0.05 and confidence intervals will be reported. This has been added to the text. |

| Reviewer 2 | Response |
| --- | --- |
| Page 4 line 89 Amend: NICE guidelines [11] on the treatment of sleepiness problems recommend that pharmacological or surgical therapy should be preferred over nonpharmacological therapy (such as behavioral stimulus methods). | We have amended this in the manuscript. |
| Page 5 lines 99-113 Redundant concept for an introduction, since then the same thing is explained in the methods. In my opinion, it is better to summarize the basic concepts in a few lines and add these things in the corresponding section. | We have moved the section below into the Materials and Methods section.    In a proof of concept and feasibility pilot [12], employing a wrist-worn tactile cue (PD-CUE) to increase swallowing regularity, 22 from 28 participants found positive benefits, with significant overall group differences on visual analogue scale self-rating of drooling frequency and severity pre-post the 4-week intervention. Early indications are that devices prove highly successful for many people with PD, but not everyone. Further work is required to test innovative cueing methods on larger populations. |
| Also, the introductory section should be shortened. | Included as part of the copyediting process agreed above. |
| Further, these reviews could be cited in the introduction as represent extensive summary of digital therapeutics and Parkinson: PMID: 32667839 and PMID: 34018047. |  |
| Page 11-12 line 244-265 I would move the "CueBand" section to the beginning, otherwise one reads the entire intervention protocol without knowing the functionality of this tool. | “CueBand” section moved to the beginning of the Materials and Methods section. |
| Page 11 line 234 - Are there also exclusion criteria? - Also, as I understand it, these criteria are only for selecting the 300 out of 3000 to be included in the comparative cueing study. How are the 3000 selected? | We have added additional text to the manuscript with our specific inclusion exclusion criteria.  With regards to the ‘selection’ of participants into either the 300-participant intervention study (Groups A/B) and the wider *free living* (Group C). We have allowed the participants to express which arm of the study they would like to participate in. However, there is an additional layer of eligibility checking on their selection.  Group A/B: must self-report as having PD, must self-report as having drooling symptoms, must declare an interest in participating in the trial. Must have 24/7 access to a compatible smartphone device with vibrotactile motor (detected by our mobile app); must provide consent to data collection.  We then randomly assign participants to either Group A/B using the process described in the manuscript.  Group C: must self-report as having PD; must declare an interest in participating in the free-living study; must have regular access to a compatible smartphone device. must provide consent to data collection.  We try to match participants with the intervention Trial first (assuming they have expressed this desire), if they do not meet any of the eligibility criteria then we check against the Free Living, if they don’t meet that then we will assign ‘no study’ and they will have access to the application without any data collection (still have the option of sharing feedback in the forms). This helps to ensure that we do not introduce any crossovers in our study population for the Intervention Trail (300).  Note, we do not send a device to anyone that is in the ‘no study’ assignment. They will be able to use the smartphone for cueing; or can still obtain a device by purchasing one from the manufacturer’s website. |
| Page 11 line 241 For patients recruited through mailing lists, or even those from the clinic, how is it determined that they have "sufficient cognitive function and manual dexterity"? | Our mailing lists were used to allow individuals to express an interest in the study and be notified when recruitment opened. They have not consented to or formally joined the study until they install the smartphone app, and complete the questionnaire within the smartphone application to participate in the study.  If they are able to perform those tasks then we will assume that they have “sufficient cognitive function and manual dexterity” as required to participate in the study. For those who are recruited in clinic we will not include those people who do not appear to have “sufficient cognitive function and manual dexterity”, and they would, in any case, be guided to complete the application themselves.  Text has been added in relation to this. |
| Page 11 line 244 It does not seem to me to be specified in the text whether the bracelet will also have an auditory signalling method in addition to the vibratory one, which may not be sufficient in some cases. | The device does not have any audio output devices that would allow it to provide such feedback to the wearer. |
